# Shared decision-making in advance care planning for persons with dementia in nursing homes: a cross-sectional study

**DOI:** 10.1186/s12877-020-01797-0

**Published:** 2020-10-02

**Authors:** Bart Goossens, Aline Sevenants, Anja Declercq, Chantal Van Audenhove

**Affiliations:** 1 LUCAS - Centre for care research & Consultancy, KU Leuven, Minderbroedersstraat 8, Postal box 5310, 3000 Leuven, Belgium; 2 Academic Center for General Practice, KU Leuven, Kapucijnenvoer 33j, Postal box 7001, 3000 Leuven, Belgium; 3 Centre for Sociological Research, KU Leuven, Parkstraat 45, Postal box 3601, 3000 Leuven, Belgium

**Keywords:** Advance care planning, Communication, Dementia, Nursing, Nursing homes, Shared decision-making

## Abstract

**Background:**

Shared decision-making provides an approach to discuss advance care planning in a participative and informed manner, embodying the principles of person-centered care. A number of guided approaches to achieve shared decision-making already exist, such as the three-talk model. However, it is uncertain whether daily practice methods in nursing home wards for persons with dementia comply with the underpinnings of this model. It is also uncertain whether professionals consider shared decision-making to be important in this context, and whether they perceive themselves sufficiently competent to practice this approach frequently.

**Methods:**

The study has a cross-sectional design, with 65 wards (46 Belgian nursing homes) participating in the study. We compared nursing home professionals’ and residents’ perspectives on the level of shared decision-making during advance care planning conversations with ratings from external raters. Residents and professionals rated the level of shared decision-making by means of a questionnaire, which included the topic of the conversation. External raters assessed audio recordings of the conversations. Professionals filled in an additional self-report questionnaire on the importance of shared decision-making, their competence in practicing the approach, and with what frequency.

**Results:**

At ward level, professionals and residents rated the average achieved level of shared decision-making 71.53/100 (σ = 16.09) and 81.11/100 (σ = 19.18) respectively. Meanwhile, raters gave average scores of 26.97/100 (σ = 10.45). Only 23.8% of residents referred to advance care planning as the topic of the conversation. Professionals considered shared decision-making to be important (x̄=4.48/5, σ = 0.26). This result contrasted significantly with the frequency (x̄=3.48/5, σ = 0.51) and competence (x̄=3.76/5, σ = 0.27) with which these skills were practiced (*P* < 0.001).

**Conclusions:**

Residents with dementia are grateful when involved in discussing their care, but find it difficult to report what is discussed during these conversations. Receiving more information about advance care planning could provide them with the knowledge needed to prepare for such a conversation. External raters observe a discrepancy between the three-talk model and daily practice methods. Training programs should focus on providing professionals with better knowledge of and skills for shared decision-making. They should also promote team-based collaboration to increase the level of person-centered care in nursing home wards for persons with dementia.

## Background

Advance care planning (ACP) is a process which enables individuals to define goals and preferences for future medical treatment and care, to discuss these goals and preferences with family and health-care providers, and to record and review these preferences if appropriate [1]. For persons with dementia, ACP is of utmost importance due to the gradual loss of decision-making capacity [2–4]. Research indicates that persons with dementia value these opportunities to increase their involvement in care choices, making them feel empowered and informed [5]. Though it is recommended to initiate ACP early, while the person with dementia still has sufficient mental capacity for being able to think about and express their preferences, its actual realization is often lacking due to both caregivers’ and families’ hesitancy to discuss the topic [1, 6–9]. Several key moments, such as the transition to a nursing home, can serve as additional opportunities to discuss ACP [10]. However, only 11.8% of persons with dementia in Belgian nursing homes have discussed their preferred care at the end of their life, having missed multiple opportunities to explore care preferences [11, 12]. Health professionals hesitate to discuss end-of-life choices, citing both individual and organizational barriers to ACP [13].

Shared decision-making (SDM) provides an approach to discuss ACP in a participative and informed manner. It is defined as a process in which both patient and healthcare professionals make decisions together, using the best available evidence [14]. SDM embodies the principles of patient-centered care, which seeks to provide high quality care by acknowledging the personhood of patients in all aspects of care, and is particularly relevant in ACP for this reason [15, 16]. Benefits of SDM include improved knowledge on health and care topics, increased participation in decision-making, reduced decisional conflict and improved confidence and coping skills [17, 18]. A number of guided approaches to SDM have been created to increase its uptake, including the three-talk model by Elwyn et al. [19, 20]. The model stipulates three steps to achieve SDM: introduce options (*choice talk*), discuss these options (*option talk*) and make a decision after exploring preferences (*decision talk*). It provides a practical, easy to teach way to train clinicians while being aligned with a more extensive conceptual model of collaborative deliberation [21].

Despite existing research underlining the importance of SDM in ACP and the availability of guided approaches such as the three-talk model, little is known about the current level of SDM during ACP conversations between nursing home residents with dementia and health professionals. Do residents with dementia consider themselves involved in the decision-making process? Does this view align with the perception of the health professional? Moreover, how do these views compare to the steps indicated by the three-talk model? Finally, do health professionals consider SDM as an important approach to ACP as indicated by literature and do they feel competent enough to implement the approach in practice? Exploring these questions might provide more insight into the reasons for the lack of SDM in practice [22].

## Aims

This study explores how health professionals and residents with dementia perceive the level of SDM during ACP conversations. In addition, professionals’ perceptions of the importance of SDM, their perceived competence and self-report about the frequency of utilizing SDM are investigated.

## Methods

### Design

This is a cross-sectional study. Data were gathered from January–July 2016. During this period, participating nursing home wards sent in at least two audio recordings of an ACP conversation. In accordance with the general definition of ACP, these recordings could include a broad range of discussions on individual goals and preferences. These conversations were compared to the three-talk model and consequently rated on the achieved level of SDM by external raters. Furthermore, when a participating nursing home ward had conducted an ACP conversation, and if both health professionals and residents with dementia were willing to complete written informed consents, they independently rated the level of SDM afterwards. They also registered information on the topic and scope of the conversation. When a resident with dementia could not provide a rating, a family member who was present during the conversation provided the information instead.

Finally, all participating health professionals filled in a self-report questionnaire on the importance of SDM, their competence in utilizing SDM and the frequency of practicing the principles of SDM in ACP conversations.

### Setting

Data were collected as part of the ‘We DECide optimized’ study, for which all 755 Flemish nursing homes (Belgium) were invited to participate. Three umbrella organizations were contacted as well to increase participation in the study. Eligible nursing homes were required to have at least one ward with people with dementia. Forty-six nursing homes (6% RR) decided to participate in the intervention, resulting in 65 wards being enlisted. For more information on the design of ‘We DECide optimized’, we refer to the study protocol [23].

### Participant characteristics

Participants in this study included health professionals, persons with dementia or family members and two external raters.

Participating health professionals were selected by the staff of the nursing home ward, with each ward selecting four to six participants. Participants were required to be care and non-care professionals, including members of management, who performed regular conversations about ACP with residents or relatives. In this way, 311 staff members were selected. They provided information on their age, sex, educational level, profession and job tenure. We also recorded whether discussing ACP was part of their routine, and if they ever received any training on SDM.

Participating wards then contacted all residents with dementia with whom an ACP conversation was scheduled between January and July of 2016. After receiving more information on the study, these persons with dementia were then asked to participate. This resulted in 42 residents participating. We recorded no information on the characteristics of the residents with dementia or their relatives.

Four external raters with a background in psychology rated the audio recordings. All raters were blind to the design of the study and did not analyze any of the data afterwards.

### Measurements

We used the Dutch version of two internationally validated self-descriptive instruments (SDM-Q, CollaboRATE) to analyze the level of SDM from the perspective of the resident and the health professional. Immediately after discussing ACP, staff members filled in the *SDM-Q-DOC*, while residents (if capable) or a family member filled in the *SDM-Q-9* [24–26]. These complementary 9-item questionnaires describe different steps of the SDM process. Items are scored on six-point Likert scales ranging from 0 (*‘completely disagree’*) to 5 (‘*completely agree’*). Residents or families also filled in *CollaboRATE* at the same time [27]. This three-question instrument allows for an additional measurement of the level of SDM from the resident’s perspective. Items are scored on 10-point Likert scales ranging from 0 (‘*no effort was made*’) to 9 (‘*every effort was made’*).

OPTION-12, an internationally validated observation method, was used to analyze the level of SDM from the perspective of the external raters [28, 29]. Two groups of two independent, blind researchers with a background in psychology each rated a random subset of the recordings after reading the OPTION training manual. The tool consists of 12 items scored on 5-point Likert scales ranging from 0 *(‘the behavior is not observed’*) to 4 (*‘the behavior is exhibited to a very high standard’*). These items reflect the different steps in the previously described three-talk model by Elwyn et al. [19, 20] and are thus used as a measurement of how the guided approach to SDM is currently practiced in the nursing home setting.

We measured professionals’ perceptions of the importance of SDM, their competence in utilizing SDM and the frequency with which they bring into practice the principles of SDM in ACP conversations by using the self-report questionnaire IFC-SDM by Ampe et al. [30]. Importance, competence and frequency were appraised in three situations: during time of admission, during crisis and during daily conversations. Health professionals scored each variable for each situation on 5-point Likert scales.

### Statistical analysis

All analyses were performed using SPSS 25.

Values at both the clinical and the ward level are provided. Guttman’s lambda-2 (λ^2^) was used as a reliability estimate for the different tests. Kappa scores were calculated when comparing scores between raters for both SDM-Q-DOC/SDM-Q-9 and OPTION-12. Additionally, intra-class correlations (ICC) were computed for OPTION-12 to estimate the inter-rater reliability between the different pairs of raters [31].

For CollaboRATE scores, the recommended top score approach was used: conversations were encoded as 1 when responses to all three items was 9 and 0 when response to at least one item was less than 9. The percentage of all code 1 conversations was then calculated, which represents the level of SDM [32]. For OPTION-12, we averaged the scores of both raters to obtain a single score, as suggested by the authors of the scale [28, 29].

## Results

### Characteristics of the professionals

The overall distribution of staff characteristics is shown in Table 1. Staff members were mostly female (87.5%) with an overall mean age of 41 (11) years. The average number of years on the job is 14 (10). ACP is part of the routine for 76.2% of staff members, especially in middle management. Less than half of the staff members (45.7%) received training on ACP. In addition, in one third of these cases, training merely consisted of the legally required program for palliative care reference persons, which is rather broad and includes limited information on ACP.

**Table 1:** Characteristics of the professionals

Characteristics		Total *N* = 311 (%)
Sex	Male	39 (12.5)
	Female	272 (87.5)
Educational status	Secondary school	43 (13.8)
	College	230 (74.0)
	University	38 (12.2)
Profession	Professionals	152 (48.9)
	- Nurse	85
	- Nursing assistant	33
	- Support roles	34
	Middle management	136 (43.7)
	- Chief nurse	72
	- Medical director	5
	- Specialist coordinator	59
	Executive management	23 (7.4)
	- Nursing home director	23
Discusses ACP frequently	Yes	237 (76.2)
	No	74 (23.8)
Previous ACP training	Yes No	142 (45.7) 169 (54.3)

### Observed level of SDM in ACP conversations

#### Residents’ and staff members’ perspectives

We received 42 fully completed versions (23.9%) of both SDM-Q-DOC, SDM-Q-9 and CollaboRATE. Either both the SDM-Q and CollaboRATE questionnaires were filled out, or neither. The main reason for not completing the questionnaires was fatigue of the resident or the relatives after the conversation. Reliability estimates reported by Guttman λ-2 showed values of 0.84, 0.96 and 0.84 for SDM-Q-DOC, SDM-Q-9 and CollaboRATE respectively.

Table 2 shows the scaled SDM-Q scores at individual and at ward level. There is a significant difference in mean total scores between professionals and residents (t = − 2.479, *P* = 0.015), with professionals consistently allocating lower scores to the level of SDM.

**Table 2:** Scaled SDM-Q-DOC (professional) and SDM-Q-9 (resident) scores by analysis level

Level	Questionnaire	N	Mean	Median	SD	Minimum	Maximum
Individual level	SDM-Q-DOC	42	71.53	72.22	16.09	37.78	100
	SDM-Q-9	42	81.11	82.22	19.18	0	100
Ward level	SDM-Q-DOC	19	71.58	68.89	13.94	50	91.11
	SDM-Q-9	19	81.50	80.74	12.69	62.22	100

Scores range 0–100

Kappa scores for single items range from −0.04 to 0.59. Both conversation partners moderately agree that the need to make a decision was clearly expressed (κ = 0.59). They differ in opinion on the extent to which treatment options were discussed (κ = − 0.04). We explored this statement further by looking at the conversation summary each party provided. Almost all professionals (41/42) mentioned ACP as the subject of the conversation, and included a number of topics discussed. In contrast, only 10/42 residents or relatives (23.8%) referred to ACP. Another 11/42 persons (26.2%) wrote down a single discussed topic, while 21/42 (50%) could not provide a topic.

The top score approach shows that 45% of residents or relatives gave a maximum score on all three items of CollaboRATE. Pearson correlation indicates a positive relation between SDM-Q-9 and CollaboRATE (r = 0.436, *P* = 0.004).

#### External raters’ perspective

The OPTION data consisted of 170 audio files from all 65 wards (100% RR). The intra-class correlation coefficients for the total score for each group of raters were 0.89 and 0.78 respectively. At the item level, there was moderately high variability within the two sets of observers: Kappa scores ranged from 0.54–0.87 and 0.49–0.85 respectively.

Individual conversations received an average score of 27.30/100 (σ = 12.73), ranging from 5.21 up to 65.63. At ward level, this resulted in average scores of 26.97 out of 100 (σ = 10.45).

Single item scores were skewed, with the majority lying between 0 (behavior is absent) and 2 (minimum skill level) (see Table 3). The behavior least demonstrated by professionals in the conversations was “assessing the resident’s preferred approach to receiving information to assist decision-making” (x̄=0.13). “Drawing attention to an identified problem that requires decision-making” received the highest average score (x̄=2.06).

**Table 3:** OPTION-12 item scores

No	Item	Mean	Median	SD
1	The clinician draws attention to an identified problem as one that requires a decision making process	2.06	2.00	0.67
2	The clinician states that there is more than one way to deal with the identified problem	1.10	1.00	0.57
3	The clinician assesses the preferred approach to receiving information to assist decision making	0.13	0.00	0.28
4	The clinician lists ‘options’, which can include the choice of ‘no action’	1.60	1.5	0.85
5	The clinician explains the pros and cons of options	0.99	1.00	0.89
6	The clinician explores the expectations (or ideas) about how the problem(s) are to be managed	1.90	2.00	0.90
7	The clinician explores the concerns (fears) about how problem(s) are to be managed	0.74	0.50	0.82
8	The clinician checks that the information has been understood	0.44	0.00	0.63
9	The clinician offers explicit opportunities to ask questions during the decision making process	0.86	0.75	0.80
10	The clinician elicits the preferred level of involvement in decision-making	1.08	1.00	1.05
11	The clinician indicates the need for a decision making (or deferring) stage	1.35	1.00	0.97
12	The clinician indicates the need to review the decision (or deferment)	1.32	1.50	1.05

Scores range 0–4, with a score ≥ 2 meaning the minimum skill level has been achieved

Clustering scores to ward level revealed that only two wards reached an average score above the minimum skill level of 50/100, scoring 53/100 and 64/100 respectively. Residents were present during 89/170 (52.4%) of the conversations. Conversations during which residents were present, correlated negatively with OPTION scores (r = − 0.246, *P* < 0.001). This means that less SDM was observed when the person with dementia attended the conversation. Discussions lasted an average of 25.70 (±19.71) minutes. Longer conversations correlated significantly with higher OPTION scores (r = 0.404, *P* < 0.001).

### Assessing views on importance, frequency and competence in shared decision-making (IFC-SDM)

The IFC-SDM was filled in by 280 professionals (90.0% RR). Of all non-respondents, 45% stated insufficient experience in discussing ACP to assess the different SDM skills. Guttman λ-2 values for the categories importance, frequency and competence were 0.95, 0.98 and 0.96.

Pearson correlation indicated a positive relationship between how important professionals considered SDM to be and how competent they felt in applying SDM (r = 0.315, *p* < 0.001). Perceived importance of SDM was also related to how frequently they participated in SDM (r = 0.278, *p* < 0.001). Finally, perceived competence was positively associated with frequency of use (r = 0.510, *p* < 0.001).

In Table 4 the IFC-SDM scores are grouped by analysis level. Nursing home staff considered SDM to be important (x̄=4.48/5, minimum item score 3/5). This result contrasts significantly with the frequency and competence with which these skills were put into practice (*P* < 0.001). SDM was considered significantly more important during crises than during daily conversations (one-way ANOVA F(2,837) = 3.90, *P* = 0.021; post-hoc Tukey mean difference 0.11 ± 0.04, *P* = 0.016). The frequency of using SDM skills and the feelings of competence did not differ between the types of conversation.

**Table 4:** IFC-SDM scores for each category by analysis level

Level	Category	Mean	Median	SD
Individual level	Importance	4.48	4.54	0.42
	Frequency	3.50	3.67	0.86
	Competence	3.76	3.89	0.50
Ward level	Importance	4.48	4.48	0.26
	Frequency	3.48	3.54	0.51
	Competence	3.76	3.78	0.27

Scores range 1–5

The highest scoring items in all three categories were “exploring residents’ preferences” and “offering the option to re-discuss decisions at a later point in time”. “Providing information on different care options”, “discussing the (dis)advantages of different care options”, and “guiding residents towards making a decision” were considered to be the least important SDM skills. These skills were also used less frequently and were associated with lower feelings of competence.

## Discussion

We compared residents’/relatives’, professionals’ and external raters’ perspectives about the use of SDM during ACP conversations. Furthermore, we measured professionals’ perceptions towards the importance of SDM, their competence in applying SDM and how frequently they practice SDM.

Overall, residents with dementia and relatives appreciated being involved in making decisions about their (health)care. However, they disagreed with professionals on which topics were addressed, and on the extent to which treatment options were discussed. Half of the persons with dementia or their relatives could not provide the topic of the conversation, with only a quarter of the residents indicating ACP as the subject of the discussion. Possibly, residents and relatives are grateful for being able to discuss their care as such, and thus give high scores, even though they remain unaware of having discussed ACP.

Our study further shows that professionals consider providing information on different care options and discussing the (dis)advantages of options as the least important items of SDM. Furthermore, external raters noted that professionals have difficulties assessing residents’ preferred approach to receiving information to participate in decision-making. Therefore, assessing residents’ need for information, as well as extensively explaining different treatment options during conversations, is crucial to discuss ACP in a person-centered way [22]. This information could be provided by the nursing home to inform residents and family members about the course of dementia and their different options [33]. Residents or relatives could also be empowered to ask for this information themselves. The ‘Ask 3 Questions’ campaign is an example of an intervention aimed at helping persons to identify what to expect and increase involvement [34].

Third, although professionals and residents’ or relatives’ ratings of the use of SDM in ACP conversations were mostly positive, the OPTION-12 scores of the external raters showed a number of shortcomings in the use of SDM compared to the three-talk model of Elwyn et al. [19, 20]. They agree that professionals are able to identify problems that require decision-making, but note that the conversations were often focused on medical aspects, leaving no time for care preferences. The topic of ACP was rarely introduced, which often resulted in anxiety when topics such as resuscitation were brought up. Checklists were frequently used, leading to broad, close-ended answers. Conversations where preferences were discussed more in-depth, scored significantly higher. Limited information giving resulted in lower SDM scores. Although residents were present in more than half of the conversations, they were rarely actively involved by the professional, which resulted in significantly lower OPTION-12 scores. This finding corresponds with other research indicating that person-centered communication is a challenge [12, 35]. The gap between external raters’ scores, professionals and relatives’ scores highlights a discrepancy in the application of SDM between the three-talk model and daily practice methods. Possibly, health professionals lack role models in how to apply SDM in their conversations [36]. Since they often are the sole person in charge of discussing ACP, it could be helpful for professionals to learn from others in other wards or organizations.

Our results indicate that future training programs in SDM should focus on both skills and knowledge development. Professionals’ perceptions towards the importance of SDM, how competent they feel in applying SDM and how frequently they participate in it, are all positively correlated. Professionals consider SDM to be an important approach to ACP. Furthermore, they feel competent in utilizing the associated skills and indicate applying these frequently, albeit less often than expected based on their importance rating. A lack of experience in conducting ACP conversations could be at the basis of this observation, indicating the need for skills training such as role-play exercises and learning from on-site role models. Regarding the need for more information on ACP, our results indicate that professionals still seem to consider ACP a topic to be discussed during crisis situations, with much less emphasis on exploring daily care preferences. This is in contrast with evidence-based recommendations [37]. Finally, our study sample shows that a wide range of health professionals is involved in ACP. To ensure that all team members are informed and work towards realizing the residents’ preferences, it is crucial that this information is exchanged in a clear and concise manner. Thus, future training programs could target interprofessional teams and emphasize collaboration.

## Strengths/limitations

We consider the inclusion of three different perspectives when assessing the use of SDM skills in ACP conversations, in addition to professionals’ self-report questionnaires, a strength of this study.

The fact that wards could select which recordings to send in for analysis possibly resulted in selection bias. Another limitation is the low response rate from residents and relatives on the SDM-Q and CollaboRATE questionnaires. We included CollaboRATE as an alternative in case fatigue would prevent residents or relatives to fill in SDM-Q. However, either both questionnaires were filled out or neither. It is possible some residents or relatives did not respond to our call for feedback because they feared reprisals by the nursing home staff, even though we provided anonymized, pre-stamped envelopes. This study does not include information on the level of cognitive impairment among residents who participated. We relied on the expertise of the professionals to decide who could or could not be included. However, since the degree of cognitive impairment could have influenced the level of engagement in conversations about ACP, as well as the responses on the CollaboRATE measures, this needs to be considered when interpreting our results. Furthermore, we have limited information on the differences and similarities between nursing homes who did participate and those who did not, limiting the generalizability of our results to the Flemish nursing home sector as a whole.

## Conclusions

Residents with dementia and their families are grateful when involved in discussing their care, but find it difficult to report what is actually discussed during these conversations. Receiving more information about ACP, or by stimulating them to ask for this information themselves, could provide them with the knowledge needed to prepare for the conversation. External raters observe a discrepancy between the three-talk model of Elwyn et al. and daily practice methods [19, 20]. We recommend a training in SDM for nursing home staff. These training programs could focus on providing staff members with knowledge on SDM, increase their skills in applying SDM and emphasize team-based collaboration to increase the level of person-centered care in nursing home wards for persons with dementia.

## Data Availability

The datasets used and/or analysed during the current study are available from the corresponding author on reasonable request.

## Abbreviations

ACP: Advance Care Planning
SDM: Shared Decision-Making

## References

[1] Rietjens JAC, Sudore RL, Connolly M, van Delden JJ, Drickamer MA, Droger M (2017). Definition and recommendations for advance care planning: an international consensus supported by the European Association for Palliative Care. Lancet Oncol.

[2] Exley C, Bamford C, Hughes J, Robinson L (2009). Advance care planning: an opportunity for person-centered care for people living with dementia. Dementia..

[3] Houben C, Spruit M, Groenen M, Wouters E, Janssen D (2014). Efficacy of advance care planning: a systematic review and meta-analysis. JAMDA..

[4] Martin R, Hayes B, Gregorevic K, Lim WK (2016). The effects of advance care planning interventions on nursing home residents: a systematic review. JAMDA..

[5] Detering KM, Hancock AD, Reade MC, Silvester W (2010). The impact of advance care planning on end of life care in elderly patients: randomized controlled trial. BMJ..

[6] Meeussen K, Van den Block L, Echteld M, Boffin N, Bilsen J, Van Casteren V, Deliens L (2012). Older people dying with dementia: a nationwide study. Int Psychogeriatr.

[7] van der Steen J, Radbruch L, Hertogh C, de Boer ME, Hughes JC, Larkin P (2014). White paper defining optimal palliative care in older people with dementia: a Delphi study and recommendations from the European Association for Palliative Care. Palliat Med.

[8] van der Steen J, van Soest-Poortvliet M, Onwuteaka-Philipsen B, Deliens L, de Boer ME, Van den Block L (2014). Factors associated with initiation of advance care planning in dementia: a systematic review. J Alzheimers Dis.

[9] Dening KH, Sampson EL, De Vries K (2019). Advance care planning in dementia: recommendations for healthcare professionals. Palliat Care.

[10] Krones T, Andorno B, in der Schmitten J, Mitchell C, Spirig R, Zaugg K (2012). Shared decision making and advance care planning - underpinnings, similarities and differences. BMJ Support Palliat Care.

[11] Dempsey D (2013). Advance care planning for people with dementia: benefits and challenges. Int J Palliat Nurs.

[12] Vandervoort A, Houttekier D, Van den Block L, van der Steen JT, Vander Stichele R, Deliens L (2014). Advance care planning and physician orders in nursing home residents with dementia: a nationwide retrospective study among professional caregivers and relatives. J Pain Symptom Manag.

[13] Scott IA, Mitchell GK, Reymond EJ, Daly MP (2013). Difficult but necessary conversations - the case for advance care planning. Med J Aust.

[14] Elwyn G, Laitner S, Coulter A, Walker E, Watson P, Thomson R (2010). Implementing shared decision making in the NHS. BMJ..

[15] Barry MJ, Edgman-Levitan S (2012). Shared decision making - the pinnacle of patient-centered care. N Engl J Med.

[16] Edvardsson D, Winblad B, Sandman PO (2008). Person-centred care of people with severe Alzheimer’s disease: current status and ways forward. Lancet Neurol.

[17] Durand MA, Carpenter L, Dolan H, Bravo P, Mann M, Bunn F, Elwyn G (2014). Do interventions designed to support shared decision-making reduce health inequalities? A systematic review and meta-analysis. PLoS One.

[18] Coulter A, Collins A (2011). Making shared decision-making a reality. No decision about me, without me.

[19] Elwyn G, Frosch D, Thomson R, Joseph-Williams N, Lloyd A, Kinnersley P (2012). Shared decision making: a model for clinical practice. J Gen Intern Med.

[20] Elwyn G, Durand MA, Song J, Aarts J, Barr PJ, Berger Z (2017). A three-talk model for shared decision making: multistage consultation process. BMJ..

[21] Elwyn G, Lloyd A, May C, van der Weijden T, Stiggelbout A, Edwards A (2014). Collaborative deliberation: a model for patient care. Patient Educ Couns.

[22] Gjerberg E, Lillemoen L, Førde R, Pedersen R (2015). End-of-life care communications and shared decision-making in Norwegian nursing homes - experiences and perspectives of patients and relatives. BMC Geriatr.

[23] Goossens B, Sevenants A, Declercq A, Van Audenhove C (2019). ‘we DECide optimised’ - Training nursing home staff in shared decision-making skills for advance care planning conversations in dementia care: protocol of a pretest-posttest cluster randomized trial. BMC Geriatr.

[24] Kriston L, Scholl I, Holzel L, Simon D, Loh A, Härter M (2010). The 9-item shared decision making questionnaire (SDM-Q-9). Development and psychometric properties in a primary care sample. Patient Educ Couns.

[25] Rodenburg-Vandenbussche S, Pieterse AH, Kroonenberg PM, Scholl I, van der Weijden T, Luyten GPM (2015). Dutch translation and psychometric testing of the 9-item shared decision making questionnaire (SDM-Q-9) and shared decision making questionnaire - physician version (SDM-Q-doc) in primary and secondary care. PLoS One.

[26] Scholl I, Kriston L, Dirmaier J, Buchholz A, Härter M (2012). Development and psychometric properties of the shared decision making questionnaire - physician version (SDM-Q-DOC). Patient Educ Couns.

[27] Elwyn G, Barr PJ, Grande SW, Thompson R, Walsh T, Ozanne EM (2013). Developing CollaboRATE: a fast and frugal patient-reported measure of shared decision making in clinical encounters. Patient Educ Couns.

[28] Elwyn G, Edwards A, Wensing M, Hood K, Atwell C, Grol R (2003). Shared decision making: developing the OPTION scale for measuring patient involvement. Qual Saf Health Care.

[29] Elwyn G, Hutchings H, Edwards A, Rapport F, Wensing M, Cheung W, Grol R (2005). The OPTION scale: measuring the extent that clinicians involve patients in decision-making tasks. Health Expect.

[30] Ampe S, Sevenants A, Coppens E, Spruytte N, Smets T, Declercq A, Van Audenhove C (2015). Study protocol for ‘we DECide’: implementation of advance care planning for nursing home residents with dementia. J Adv Nurs.

[31] Landers RN (2015). Computing intraclass correlation (ICC) as estimates of interrater reliability in SPSS. The Winnower.

[32] Barr P, Thompson R, Walsh T, Grande SW, Ozanne E, Elwyn G (2014). The psychometric properties of CollaboRATE: a fast and frugal patient-reported measure of the shared decision-making process. J Med Internet Res.

[33] Arcand M, Brazil K, Nakanishi M, Nakashima T, Alix M, Desson J (2013). Educating families about end-of-life care in advanced dementia: acceptability of a Canadian family booklet to nurses from Canada, France, and Japan. Int J Palliat Nurs.

[34] Shepherd HL, Barratt A, Trevena LJ, McGeechan K, Carey K, Epstein RM (2011). Three questions that patients can ask to improve the quality of information physicians give about treatment options: a cross-over trial. Patient Educ Couns.

[35] Couët N, Desroches S, Robitaille H, Vaillancourt RD, Leblanc A, Turcotte S (2015). Assessments of the extent to which health-care providers involve patients in decision making: a systematic review of studies using the OPTION instrument. Health Expect.

[36] Godolphin W (2009). Shared decision-making. Healthc Q.

[37] Piers R, Albers G, Gilissen J, De Lepeleire J, Steyaert J, Van Mechelen W (2018). Advance care planning in dementia: recommendations for healthcare professionals. BMC Palliat Care.

